# Supplementary material for: Lateralized alpha oscillations are irrelevant for the behavioral retro-cueing benefit in visual working memory
Source: PeerJ. 2020 Jun 23;8:e9398. doi: 10.7717/peerj.9398 (PMC7319032; doi:10.7717/peerj.9398)
Supplement: Supplemental Information 1 — (S1) Comparison of classification accuracy with a conventional set of electrodes. (S2) Alternative analysis of the correlation between individual behavioral retro-cueing benefits and average alpha oscillation. (S3) Interaction of memory bias and target-distractor similarity. (S4) Reliability of lateralization and behavioral measures. [file peerj-08-9398-s001.pdf]

Supplemental Analyses

Lateralized Alpha Oscillations are Irrelevant for the Behavioral Retro-Cueing Benefit in Visual Working Memory

Wanja Mössing[1, 2] and Niko A. Busch[1, 2]

[1]Institute of Psychology, University of Münster, Münster, Germany
[2]Otto Creutzfeldt Center for Cognitive and Behavioral Neuroscience, University of Münster, Münster, Germany

S1: COMPARISON OF CLASSIFICATION PERFORMANCE

Using a correlation based classification, this paper employs a comparably unconventional method of quantifying alpha lateralization. To assure that this method indeed decodes more information from the EEG signal than more classical approaches, we compared the performance of the classifier using all electrodes (as is reported in the article) against a reduced set of typically used electrodes (i.e., the electrodes analyzed in Schneider et al., 2019). Specifically, we located the electrodes in our montage that were closest to PO7/8, PO3/4, P7/8, and P5/6 in a standard 10–20 montage. Based on this reduced set of eight electrodes, we then computed difference topographies and trial-wise template correlations analogous to the all-channel classification described in the main article. Figure S1 shows the outcome of this analysis. As is clearly visible, all-electrodes classification decodes significantly more information than the typically used set of electrodes. A paired two-sided t-test of and an analogous Bayes factor present strong evidence in favor of the all-electrodes classification.

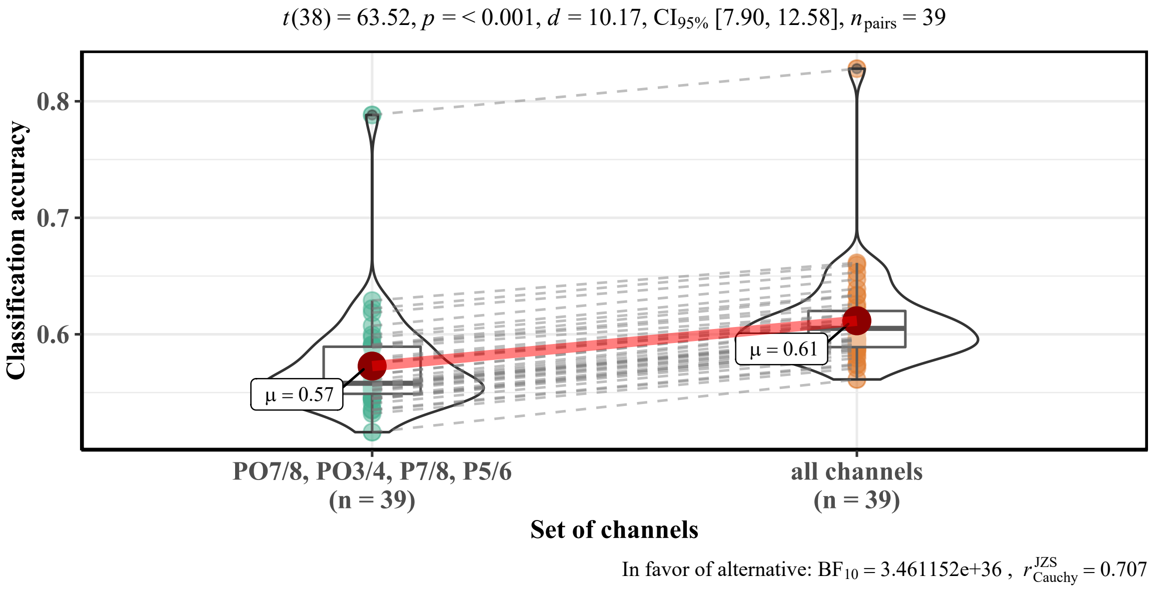

Figure S1. Comparison of individual classification accuracy using the all-electrode classification employed in this article (orange distribution), with the classification accuracy using a typically analyzed set of electrodes (see Schneider et al., 2019). The subtitle shows the result of a parametric dependent-samples two-sided t-test comparing the accuracy scores of all participants between the two classifiers. An analogous Bayes factor analysis is reported at the bottom of the plot. μ represents the mean accuracy across participants.

S2: CORRELATION OF ALPHA LATERALIZATION AND RETRO-CUE BENEFIT

In the main analysis, we quantified alpha-band lateralization using a classification-based approach. In order to compare our results to a simpler and more "classical" way of quantification, we additionally computed correlations between alpha lateralization and behavioral retro-cue benefits. Specifically, per participant, we computed retro-cue benefits as the differences between parameter estimates (κ, μ, g) for retro-cued and for not cued trials (e.g., $\kappa_{(i,\ retro-cue)} - \kappa_{(i,\ no-cue)})$[1]. Lateralized alpha power was quantified as the mean power (*pwc*) across the electrodes and time points involved in the significant cluster[2] (posterior electrodes; 500–1000 ms following the retro-cue; see results section). To test for an association between lateralized alpha power and the behavioral retro-cue benefits, we then tested Pearson correlations between each of the parameter estimates and lateralized alpha power, across participants. In addition, we computed analogous bayesian correlation tests. The results are qualitatively similar to those obtained via the classification method. Figure S2 visualizes the correlations for each parameter. One participant had a lateralization value that was more than five standard errors away from the average and was therefore excluded from this analysis.

The analysis did not yield any significant correlation between alpha lateralization and behavioral retro-cue benefits (all $p > .36$; all $|r| < .15$). Moreover, Bayes factor analysis indicates moderate evidence against correlations for all three parameters ($\kappa : BF_{01} = 3.91$; $g : BF_{01} = 2.75$; $\mu : BF_{01} = 2.82$). Thus, this analysis confirms and complements the results of the analysis reported in the main article.

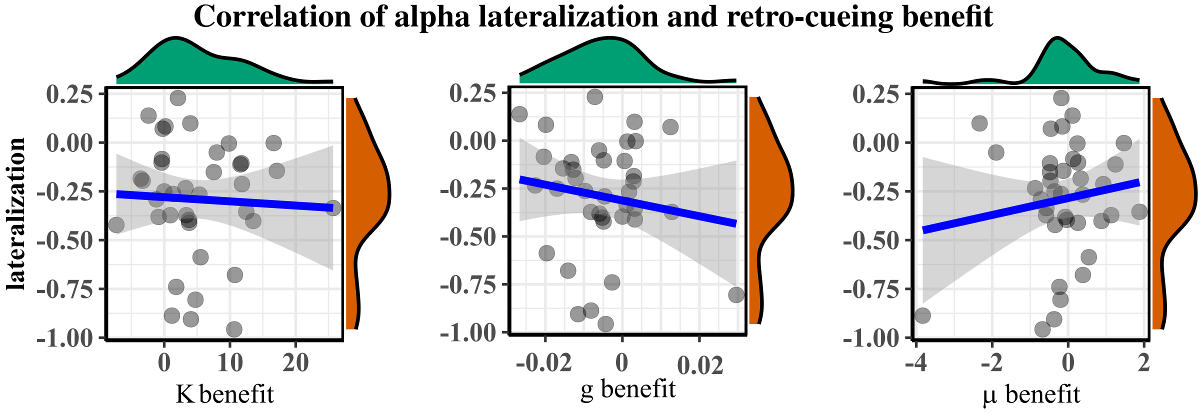

Figure S2. Correlations between individual lateralized alpha power and retro-cueing benefit. Panels display the lateralized alpha power plotted against the retro-cueing benefit for each parameter of a standard mixture model, across participants. Marginals show density estimates of the distribution of each of the two variables. Blue lines represent a linear fit with shaded 95% confidence intervals.

---

[1] Note that the analysis reported in the main article uses *cue* as a predictor in a linear mixed effects model (LMM), thereby testing the effects of retro-cues and alpha lateralization in a single model. The nature of the simpler, correlational analysis reported here, requires a single dependent variable (memory performance) per participant. Therefore, the quantification of the dependent variable differs slightly from the main analysis, as parameter estimates are collapsed over the levels of the factor "cue".

[2] For each participant, power was transformed to a dB scale by computing baseline corrected power *pwc* as:

$$pwc_{t,f,i} = 10 * log_{10}(\frac{pw_{t,f,i}}{bsl_{f,i}}),$$

where $pw_{t,f,i}$ is raw power at each time point t, frequency f and trial i, and $bsl_{f,i}$ is power averaged across the baseline interval for each f and i. Thereby, *pwc* highlights the relative change elicited by the retro-cue and attenuates effects of earlier stimulation.

S3: MEMORY BIAS AND TARGET-DISTRACTOR SIMILARITY

For the purpose of this study, it was important to show that responses are generally biased by the irrelevant distractor. As we show in the main analysis, reported orientations were, on average, biased towards the distractor's orientation (attractive bias). While this is in line with several other studies showing an attractive bias (e.g., Rademaker et al., 2015; Wildegger et al., 2015), others found a repulsion for within-trial distractors instead (e.g., Czoschke et al., 2019; Sweeny et al., 2011, see also Discussion). Although the current study was not specifically designed to compare these different types of biases, we would like to foster the discussion by providing a more detailed analysis of this bias. This analysis was motivated by Bae & Luck's (2020) finding that repulsion occurs between similar items, while attraction occurs between dissimilar items. To this end, we computed the difference angle between the target and distractor orientations and subsequently grouped each participant's trials into six bins with boundaries at -121, -60, -30, 0, 30, 60, and 121 degree difference (note that 121 degrees was the maximal possible difference in this study). We then fitted mixture models for each participant and bin, in order to obtain bin-wise bias estimates. Figure S3 shows the resulting bias estimates across bins averaged across participants. The plot shows a clear attractive bias across most target-distractor differences with no evidence for repulsion when target and distractor had similar orientations, similar to results reported by Wildegger et al. (2015).

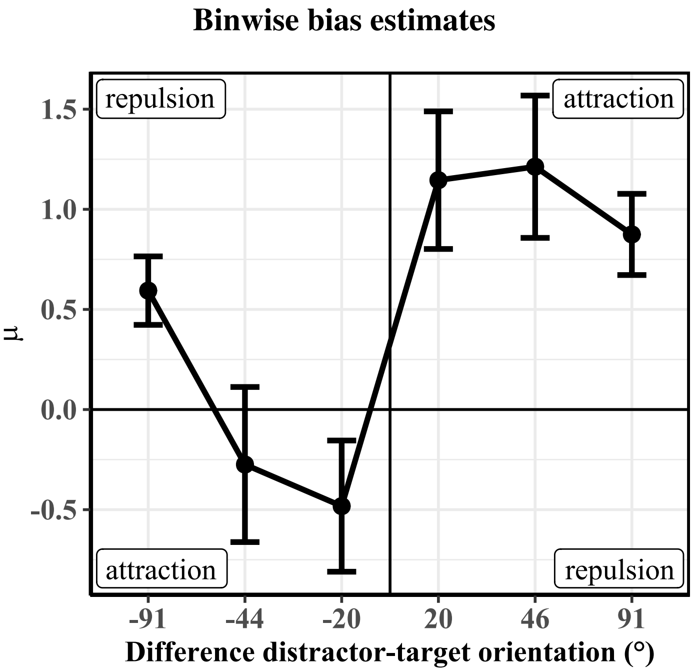

Figure S3. Binwise bias estimates. X-axis represents mean differences between target and distractor orientation; numbers indicate the bins' central orientation difference. Error bars show Cosineau-Morey (Morey, 2008) within-participants standard errors. Negative values on the y-axis indicate a counterclockwise bias, and negative values on the x-axis show trials in which the distractor was oriented counterclockwise with respect to the target.

S4: RELIABILITY OF LATERALIZATION AND BEHAVIORAL MEASURES

Our main analysis includes a correlation of alpha-band lateralization and behavioral performance across participants. It is important to demonstrate that all measures show sufficient across-participants variability, and that this variability is indicative of reliable inter-individual differences rather than measurement error. To this end, we computed, for alpha-band lateralization and for each mixture model parameter, the correlation between the first and second recording session across participants (Figures S4A & S4B). For each correlation, we computed parametric and bayesian tests. One participant had an extraordinarily high guess-rate and was therefore excluded from the correlation analysis for guess-rate. With the exception of guess-rate in the no-cue condition, all correlations were significant (see Tables S4A & S4B), in line with Bayes factors indicating strong evidence for a correlation between sessions. Thus, this analysis demonstrates that alpha-band lateralization and parameter estimates were variable across participants, and reliable within participants, thereby validating the analysis of correlations between lateralization and behavior.

Parameter	Cue	t	df	$r_{Pearson}$	p_{holm}	Bayes factor
κ	retro-cue	9.95	37	.85	$< .001$	$BF_{10} = 1275898364$
κ	no-cue	9.55	37	.84	$< .001$	$BF_{10} = 458777532$
g	retro-cue	3.24	36	.48	.02	$BF_{10} = 17.94$
g	no-cue	1.86	36	.30	.43	$BF_{10} = 1.14$
μ	retro-cue	3.62	37	.51	.003	$BF_{10} = 44.75$
μ	no-cue	3.90	37	.54	$< .001$	$BF_{10} = 91.46$

Table S4A. Correlations between the first and second recording session across subjects, separately for each mixture model parameter and cueing condition with corresponding parametric and bayesian tests. BF_{10} indicates evidence in favor of H_1 over H_0.

Probed Hemifield	Cue	t	df	$r_{Pearson}$	p_{holm}	Bayes factor
left	retro-cue	6.60	37	.74	$< .001$	$BF_{10} = 158628.1$
right	retro-cue	8.45	37	.81	$< .001$	$BF_{10} = 25869807$
left	no-cue	6.61	37	.74	$< .001$	$BF_{10} = 164123.4$
right	no-cue	8.90	37	.83	$< .001$	$BF_{10} = 85433251$

Table S4B. Correlations between the alpha lateralization of the first and second recording session across subjects, separately for probed hemifield and cueing condition with corresponding parametric and bayesian tests. BF_{10} indicates evidence in favor of H_1 over H_0.

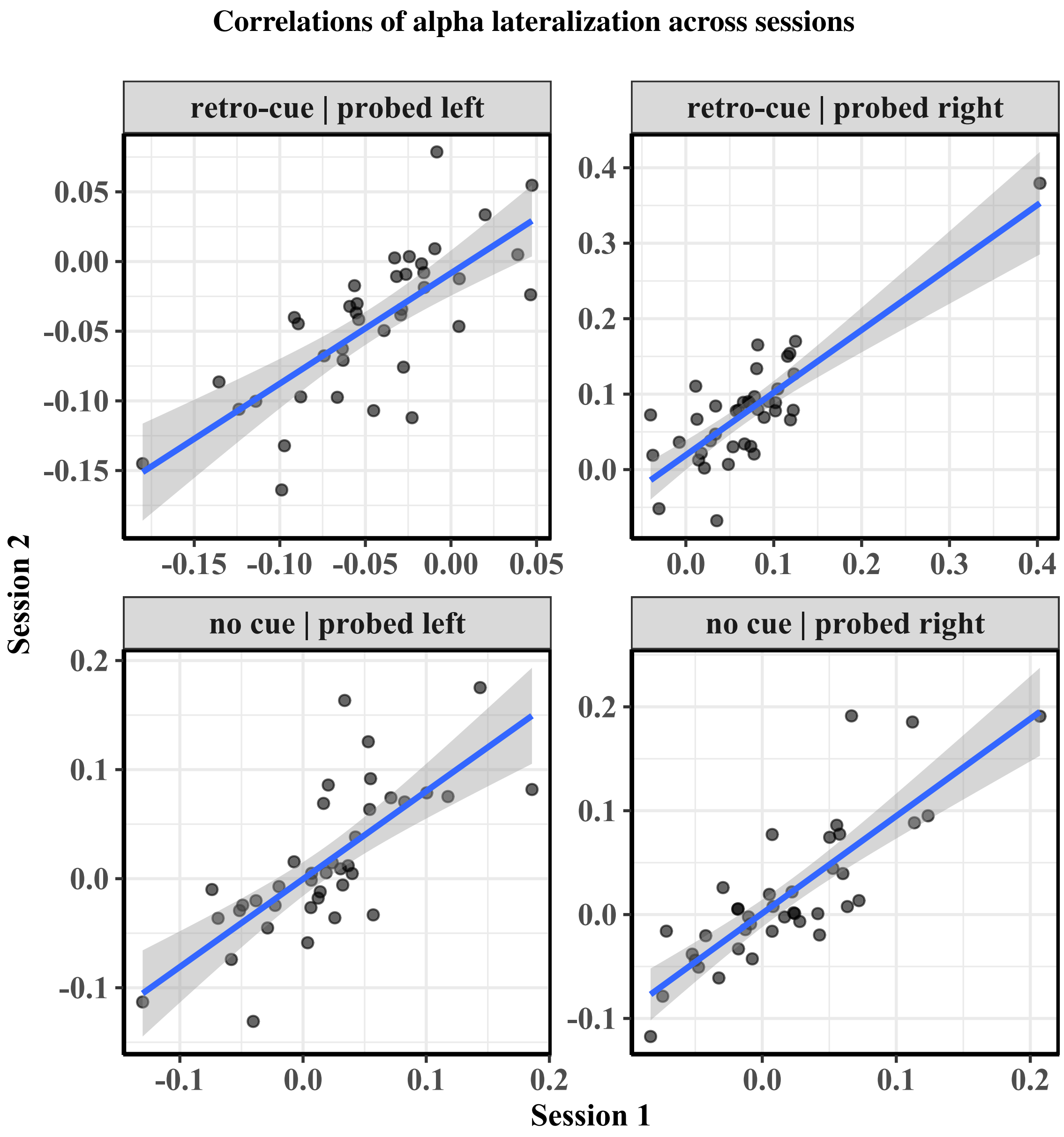

Figure S4A. Correlations between the first and second recording session across participants, separately for probed hemifield (columns), and for cued (top row) and non-cued (bottom row) trials. Blue lines represent a linear fit with shaded 95% confidence intervals.

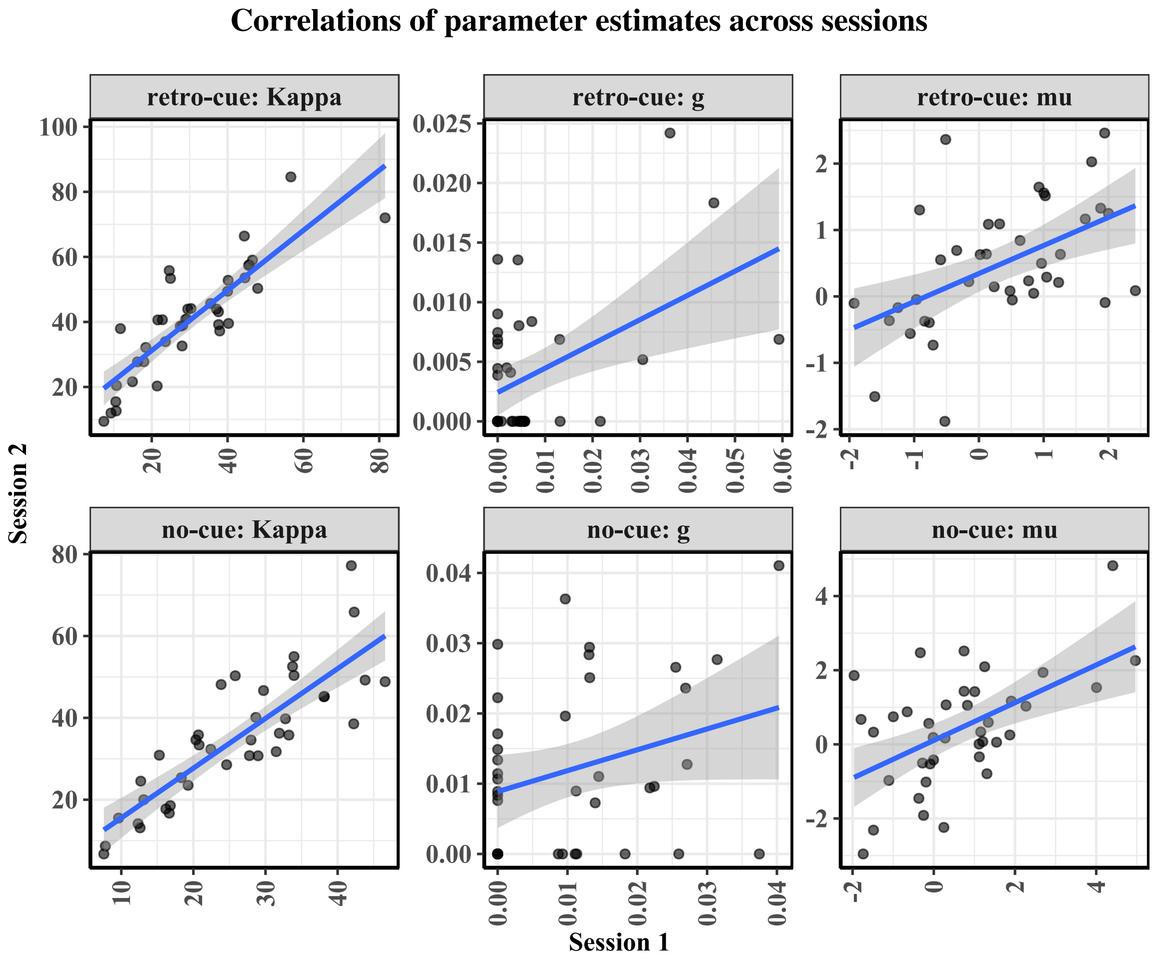

Figure S4B. Correlations between the first and second recording session across participants, separately for each mixture model parameter (columns), and for cued (top row) and non-cued (bottom row) trials. Blue lines represent a linear fit with shaded 95% confidence intervals.
