# Supplementary figures and images for: Lateralized alpha oscillations are irrelevant for the behavioral retro-cueing benefit in visual working memory
Source: PeerJ. 2020 Jun 23;8:e9398. doi: 10.7717/peerj.9398 (PMC7319032; doi:10.7717/peerj.9398)

## International 10/20

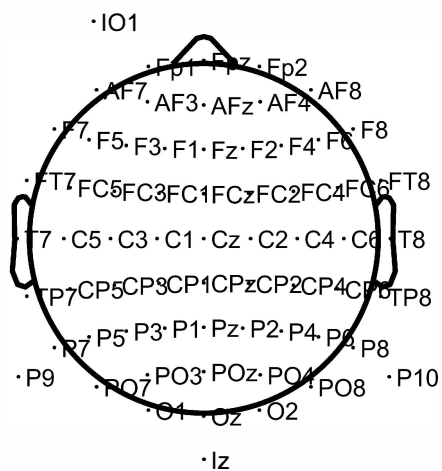

## Easycap M34

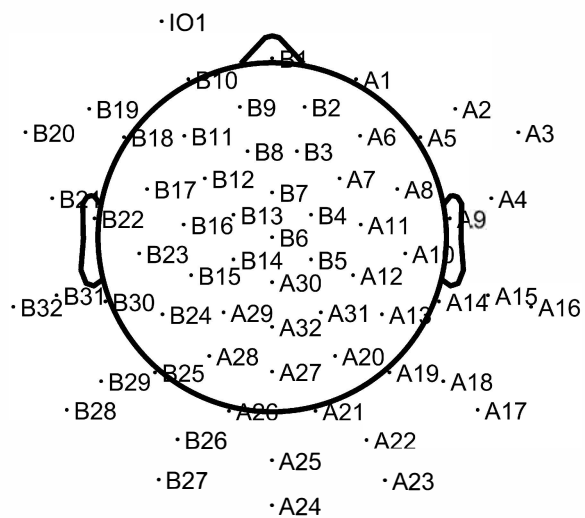

Supplement: Supplemental Information 2 — Top: International 10/20 EEG montage. Bottom: Custom "Easycap M34" montage with equidistant positioning of electrodes. The scales of both plots are matched. [file peerj-08-9398-s002.pdf]
